# Time for a standardized diagnostic response test in patients with chronic inflammatory demyelinating polyradiculoneuropathy?

**DOI:** 10.1002/brb3.3256

**Published:** 2023-09-24

**Authors:** Fabian Szepanowski, Benedikt Schoser, Anne K. Mausberg, Christoph Kleinschnitz, Hans‐Peter Hartung, Mark Stettner

**Affiliations:** ^1^ Department of Neurology Essen University Hospital, University Duisburg‐Essen Essen Germany; ^2^ Center for Translational Neuro‐ and Behavioral Sciences (C‐TNBS) University of Duisburg‐Essen Essen Germany; ^3^ Department of Neurology Friedrich‐Baur‐Institute, Ludwig‐Maximilians‐University Munich Munich Germany; ^4^ Department of Neurology Medical Faculty, Heinrich‐Heine‐University Düsseldorf Düsseldorf Germany; ^5^ Brain and Mind Center University of Sydney Sydney New South Wales Australia; ^6^ Department of Neurology Palacky University Olomouc Olomouc Czechia

**Keywords:** CIDP, dosing, efficacy, intravenous immunoglobulin, therapy

## Abstract

Standardized pharmacological response tests are important and established diagnostic tools in the field of neurology. However, regarding therapeutic responses to intravenous immunoglobulins (IVIg) in CIDP, neither a definition of therapeutic response has been established, nor a response test has been suggested so far. Here we suggest a practical clinical approach which is supported by current literature in the field. An established standardized IVIg response test could avoid prolonged therapy without benefit for the patient and ensure a timely therapy switch or treatment escalation if required. This approach would also be advantageous due to the global scarcity of plasma derivatives as a human resource and could be the foundation to be adjusted and improved by subsequent studies.

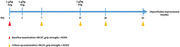

1

Standardized pharmacological response tests are established diagnostic tools in the field of neurology, for example, the L‐Dopa test for Parkinson's disease (D'Costa et al.,^1995^) or the Tensilon test for myasthenia gravis (Osserman & Kaplan, 1952; Van Dyk & Florence, 1980) have been shown to hold high diagnostic value. Response tests for neuroimmunological diseases have proven more difficult to establish, primarily due to the fact that immunosuppressive or immunomodulatory effects as well as the clinical response usually occur with delay.

In contrast to other immune‐mediated conditions, chronic inflammatory demyelinating polyradiculoneuropathy (CIDP) patients often experience objectifiable improvement in their paresis and/or sensory symptoms shortly after the initiation of therapy (van Doorn, 2005) and some even with end‐of‐dose deterioration in dependency on subsequent therapies. The guidelines on diagnosis and treatment of CIDP have already taken into account the relevance of the individual therapeutic response as a diagnostic aid and integrated therapeutic response as a “supporting criterion” into the 2021 revised criteria (Van den Bergh et al., 2021). However, a standardized response test has not been established yet.

The recently published “Progress in CIDP (ProCID) study,” which investigated the efficacy and safety of a 10% intravenous immunoglobulin (IVIg) in a three‐dose regimen for patients with active CIDP in a prospective, double‐blind, randomized multicenter design, provides valuable data regarding the development of a response test (Cornblath et al., 2022). Based on the highest response rates using an induction dosage of 2 g/kg body weight followed by maintenance doses every 3 weeks, such a dose seems efficient and safe for a diagnostic response test. The study showed that the individual response to IVIg was usually detected within 6−8 weeks. Continuing treatment beyond this timeframe to await a response may not be clinically useful. Even after only the induction therapy (at 3 weeks), the study found that 56% of all patients and 62% of responders showed an improvement of ≥1 adjusted inflammatory neuropathy cause and treatment (INCAT) score point. After 6 weeks, almost all responders achieved this positive response.

Furthermore, results from this study support the use of IVIg, as opposed to corticosteroids, for such diagnostic response testing because the majority of prior corticosteroid responders (87.1% of the overall group) also responded to IVIg. The limitation of the study ‐ the restriction of enrollment to patients with a previous and/or current response ‐ was actually beneficial for the evaluation of a diagnostic response test. The fact that the study population consisted of previous therapeutic responders is reflected by the high IVIg responder rates (92% in the 2.0 g/kg group), which were higher compared to other large CIDP studies (ICE 54% [Hughes et al., 2008], PRIMA 61% [Leger et al., 2013], and PRISM 76% [Nobile‐Orazio et al., 2020]). The study is, therefore, an unintended evaluation of a response behavior in an artificial responder group. The fact that no serious side effects such as thromboembolic events or hemolysis occurred in the study furthermore supports a regimen of two doses of 2 g/kg body weight.

As a standardized diagnostic response test in patients with CIDP, we suggest three doses of IVIg, the first two (day 1 and day 2) using 1 g/kg body weight and the third dose 3 weeks later using 2 g/kg body weight. The adjusted INCAT disability scale, grip strength, and I‐RODS have been evaluated in clinical studies to detect treatment response (Allen et al., 2021) and should be assessed as baseline scoring and be followed up on day 14 and on day 28 (Figure 1). The test could be considered positive if one of the objectifiable parameters shows improvement.

**FIGURE 1:**
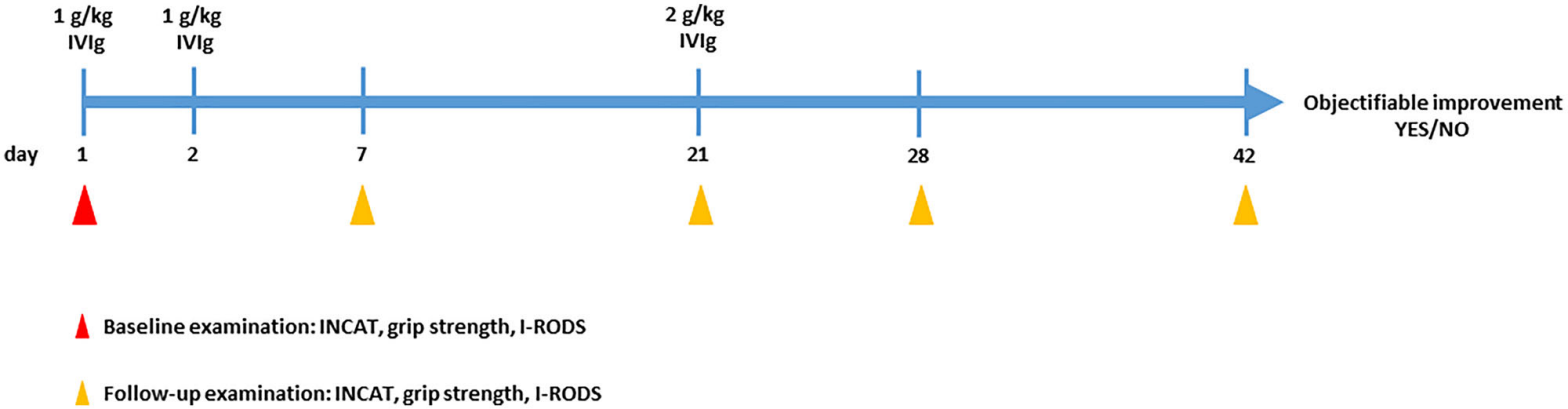
Treatment scheme for the proposed “therapeutic response test.”

We cannot exclude that a fraction of “late responders” shows treatment response up to 6 months after treatment initiation, as a recent open‐label, single‐arm study suggested (Nobile‐Orazio et al., 2020). The higher dose of IVIg in our response test appears likely to ensure a higher percentage of responders within the first weeks of treatment due to the greater availability of immunoglobulins in a shorter time frame as shown by dose−response profiles in previous trials.

An established standardized IVIg response test could avoid prolonged therapy without benefit for the patient and ensure a timely therapy switch or treatment escalation if required. This approach would also be advantageous due to the global scarcity of plasma derivatives as a human resource and may provide the foundation to be adjusted and improved by subsequent studies.

## AUTHOR CONTRIBUTIONS

MS conceptualized the manuscript. MS and FS wrote the manuscript. All authors reviewed the manuscript for intellectual content and critically revised the manuscript.

2

### PEER REVIEW

The peer review history for this article is available at https://publons.com/publon/10.1002/brb3.3256
